# Side Effects of Radiographic Contrast Media: Pathogenesis, Risk Factors, and Prevention

**DOI:** 10.1155/2014/741018

**Published:** 2014-05-11

**Authors:** Michele Andreucci, Richard Solomon, Adis Tasanarong

**Affiliations:** ^1^Nephrology Unit, Department of “Health Sciences”, Campus “Salvatore Venuta”, “Magna Graecia” University, Loc. Germaneto, 88100 Catanzaro, Italy; ^2^University of Vermont College of Medicine, Fletcher Allen Health Care, Burlington, VT, USA; ^3^Nephrology Unit, Department of Medicine, Faculty of Medicine, Thammasat University, Rangsit Campus, Khlong Luang, Pathum Thani 12121, Thailand

## Abstract

Radiocontrast media (RCM) are medical drugs used to improve the visibility of internal organs and structures in X-ray based imaging techniques. They may have side effects ranging from itching to a life-threatening emergency, known as contrast-induced nephropathy (CIN). We define CIN as acute renal failure occurring within 24–72 hrs of exposure to RCM that cannot be attributed to other causes. It usually occurs in patients with preexisting renal impairment and diabetes. The mechanisms underlying CIN include reduction in medullary blood flow leading to hypoxia and direct tubule cell damage and the formation of reactive oxygen species. Identification of patients at high risk for CIN is important. We have reviewed the risk factors and procedures for prevention, providing a long list of references enabling readers a deep evaluation of them both. The first rule to follow in patients at risk of CIN undergoing radiographic procedure is monitoring renal function by measuring serum creatinine and calculating the eGFR before and once daily for 5 days after the procedure. It is advised to discontinue potentially nephrotoxic medications, to choose radiocontrast media at lowest dosage, and to encourage oral or intravenous hydration. In high-risk patients N-acetylcysteine may also be given.

## 1. Introduction

Radiographic contrast media are a group of medical drugs used to improve the visibility of internal organs and structures in X-ray based imaging techniques such as radiography and computed tomography (CT). The currently used contrast media are based on the chemical modification of a 2,4,6-tri-iodinated benzene ring and are indispensable in the practice of radiology, for both diagnostic and therapeutic purposes. Iodine-based contrast media are usually classified as ionic or nonionic and as monomeric and dimeric and are commonly used to visualize vessels, tissues, organs, and the urinary tract. They are helpful in differentiating between normal and pathological areas. They are usually safe, and adverse effects are generally mild and self-limited.

Side effects of radiographic contrast media range from a mild inconvenience, such as itching, to a life-threatening emergency [1]. Contrast-induced nephropathy (CIN) is a well known adverse reaction associated with the use of intravenous or intra-arterial contrast material. Other forms of adverse reactions include delayed allergic reactions, anaphylactic reactions, and cutaneous reactions.

Previous allergic reactions to contrast material increase the risk of developing adverse reactions to contrast agents. Pretreatment of patients who have such risk factors with a corticosteroid and diphenhydramine decreases the chance of allergic reactions, including anaphylaxis or life-threatening emergency. Of the former either prednisone (50 mg orally, 13, 7, and 1 h before contrast injection), or hydrocortisone (200 mg intravenously, 1 h before contrast injection), or methylprednisolone (32 mg orally, 12 and 2 h before contrast media injection) is used. Diphenhydramine (50 mg intravenously/intramuscularly/orally, 1 h before contrast injection) is also used [2].

Awareness of different risk factors and screening for their presence before the use of contrast agents allow for early recognition of adverse reactions and their prompt treatment.

The most important adverse effects of contrast media include hypersensitivity reactions, thyroid dysfunction, and contrast-induced nephropathy [3].

## 2. Hypersensitivity Reactions to Radiographic Contrast Media

Mild hypersensitivity reactions (incidence <3%) consist of immediate skin rashes, flushing or urticaria pruritus, rhinorrhea, nausea, brief retching, and/or vomiting, diaphoresis, coughing and dizziness; moderate to severe (incidence <0.04%) reactions include persistent vomiting, diffuse urticaria, headache, facial edema, laryngeal edema, mild bronchospasm or dyspnea, palpitations, tachycardia or bradycardia, abdominal cramps, angioedema, coronary artery spasm, hypertension or hypotension, life-threatening cardiac arrhythmias (i.e. ventricular tachycardia), overt bronchospasm, laryngeal edema, cardiac failure and loss of consciousness, pulmonary edema, seizures, syncope. Mortality is less than one death per 100000 patients [3].

Asthma, history of multiple allergies, and therapy with beta blockers increase the risk of bronchospasm.

As soon as a reaction occurs, infusion of the contrast media should be ceased immediately and treatment with antihistamine immediately started. Bronchospasm and wheezing, laryngospasm and stridor or hypotension should be treated immediately with adrenaline, intravenous fluids, and oxygen, in addition to antihistamines with or without hydrocortisone [3].

Hypersensitivity reactions to contrast media include both Ig E and non-Ig E-mediated anaphylaxis, with activation of mast cells, coagulation, kinin and complement mechanisms, inhibition of enzymes, and platelet aggregation [3].

Delayed adverse reactions to radiographic contrast media are usually cutaneous (reported incidence varies from 1% to 23%) and include rash, skin redness, and skin swelling, sometimes associated with nausea, vomiting, and dizziness, that begin 1 hour or longer (usually 6–12 hours) after the administration of the contrast agent; they are usually mild and non-life threatening (sometimes can be moderate to severe) and often not brought to the attention of the radiologist and are ascribed to other causes [4]. Since patients are generally discharged from the radiology department within half an hour of contrast administration, these reactions are rarely observed by the radiologist supervising the contrast administration. Adverse delayed cutaneous events have been noted significantly (*P* < 0.05) more often with a dimeric nonionic agent (16.4%) than with a monomeric nonionic contrast agent (9.7%) [5]. Cutaneous reactions vary in size and presentation but are usually pruritic. For the most part, these reactions are self-limited and symptoms can be treated with corticosteroid creams.

In a prospective study comparing a group of patients undergoing computed tomography (CT) with iohexol and another group undergoing CT without contrast media, delayed cutaneous adverse reactions were significantly more frequent (*P* < 0.001) in the iohexol group (14.3%) than in the control group (2.5%) [4]. Similarly, in two prospective studies there was a significantly higher rate of rash following the intra-arterial utilization of iodixanol (Visipaque 320, 12.2%, and 10.4%) than with either the monomer iopamidol (Niopam 300) or the ionic dimer ioxaglate (Hexabrix) (2.7%–4.2%) [6, 7].

The pathophysiology of delayed cutaneous reactions is speculative but likely represents a spectrum of T cell-mediated delayed hypersensitivity [4].

## 3. Contrast-Induced Thyroid Dysfunction

Iodinated contrast media exposure may be associated with development of either hyperthyroidism or hypothyroidism, presumably due to the effect of free, biologically active iodide ions present in the contrast media preparation. It is possible that long-term storage and exposure to light may lead to photolytic degradation of contrast media and hence an increased concentration of free iodine in solution [8].

Iodine is the important element used in contrast media that possesses high-contrast density. A dose of contrast media used in typical radiological procedure contains about 13500 *μ*g of free iodide [9] and 15 to 60 g of bound iodine [9, 10] that may be liberated as free iodide in the body [9, 11]. This is actually an acute iodide load of 90 to several hundred thousand times the recommended daily intake of iodide (150 *μ*g) [12]. The normal response to a high iodine load is the acute Wolff-Chaikoff effect, a rapid inhibition of thyroid hormone synthesis and release [13]. Following several days of continued exposure to high iodine levels, there is an escape from the acute Wolf-Chaikoff effect, mediated by downregulation of the sodium iodide transporter (NIS), which transports iodine into the thyroid, and normal thyroid hormone production resumes [14]. Failure of the acute Wolff-Chaikoff effect results in iodine-induced hyperthyroidism, or the Jod-Basedow phenomenon. Failure to escape from the acute Wolff-Chaikoff effect results in iodine-induced hypothyroidism [15–17].

Iodinated contrast-induced thyrotoxicosis is relatively rare. Patients with Graves' disease and multinodular goitre are at increased risk, and those with thyrotoxicosis should receive iodinated contrast media only with close monitoring since patients with preexisting hyperthyroidism may develop a thyroid crisis [3]. On the other hand, iodine-induced thyrotoxicosis following contrast radiography has been found in 7 of 28 cases of hyperthyroidism seen at a geriatric hospital [18]. Other studies have demonstrated the occurrence of hyperthyroidism following nonionic contrast radiography [19, 20].

Individuals with underlying Hashimoto's thyroiditis or other autoimmune thyroid diseases and those with a history of partial thyroidectomy are at particular risk for the development of iodine-induced hypothyroidism. It has been demonstrated that iodine-containing contrast media (for coronary angiography or CT; iodine dose range from 300 to 1221 mg of iodine per kilogram) can transiently induce subclinical hypothyroidism even in euthyroid patients [21].

Only few studies have been done on the possible association between contrast media exposure and subsequent functional derangements of thyroid. Recently a nested case-control study was performed to assess the association between the exposure to contrast media and incident thyroid dysfunction, using a database of patients receiving care at Brigham and Women's Hospital and at Massachusetts General Hospital in Boston, Massachusetts [17]. In this study incident hyperthyroidism was defined as a thyrotropin level, at follow-up, below the normal range and incident hypothyroidism as a thyrotropin level above the normal range; incident overt hyperthyroidism was defined as a follow-up thyrotropin level ≤0.1 mIU/L and incident overt hypothyroidism as a follow-up thyrotropin level >10 mIU/L based on evidence that such levels are associated with cardiovascular morbidity and mortality and are less likely to be due to nonthyroidal illness. The study demonstrated a significant association between iodinated contrast media exposure and subsequent development of incident hyperthyroidism, incident overt hyperthyroidism, and incident overt hypothyroidism. However, no association was found between contrast media exposure and incident hypothyroidism [17].

Iopanoic acid and ipodate, iodinated contrast media previously used for cholecystography, are potent inhibitors of the conversion of thyroxine (T4) to triiodothyronine (T3), the bioactive thyroid hormone. They were occasionally used therapeutically in hyperthyroid patients, for example, to rapidly correct severe hyperthyroidism prior to thyroidectomy [22]. However, these agents are now used infrequently and are no longer marketed in the United States.

## 4. Contrast-Induced Nephropathy

When radiographic contrast media are injected intravenously or intra-arterially, they pass from the vascular compartment through capillaries into the extracellular space. They are eliminated almost entirely by glomerular filtration, concentrated in the tubular lumen by water tubular reabsorption, thereby visualizing the urinary tract.

The use of contrast media may lead to kidney dysfunction, especially in patients with preexisting renal impairment and in those with diabetes. Contrast-induced nephropathy (CIN) or contrast-induced acute kidney injury (CI-AKI) is therefore an iatrogenic disease and has become a significant source of hospital morbidity and mortality. Several years ago it was indicated as the third leading cause of hospital-acquired acute renal failure (after surgery and hypotension) accounting for 12% of all cases [23]. It has been stated that it occurs in up to 5% of hospitalized patients who exhibit normal renal function prior to introduction of contrast [24]. For outpatients, the risk for CIN, particularly in patients with creatinine clearance >45 mL/min per 1.73 m^2^, seems to be extremely low (approximately 2%) [25]. It has been stated that CIN is not common in patients with normal preexisting renal function; rather, it occurs more frequently in patients with renal impairment and is possibly exacerbated when the impairment is due to diabetic nephropathy [26].

We may define CIN as acute renal failure occurring within 24–72 hrs of exposure to intravascular radiographic contrast media that cannot be attributed to other causes. It is commonly a nonoliguric and asymptomatic transient decline in renal function, generally occurring within 24 hrs of contrast administration, usually peaking on the third to fifth day, and returning to baseline within 10–14 days. The impairment of renal function is usually mirrored by an absolute (0.5 mg/dL or greater) or relative (by 25% or greater) increase in serum creatinine from baseline [27, 28]. Variation in serum creatinine levels after contrast media has been interpreted as indicating nephrotoxicity even though such variation may occur even without contrast media administration [29]. Thus, the incidence of CIN would have been overestimated because of fluctuations in serum creatinine level that may occur naturally or in response to acute medical instability [26]. For this reason it is better to consider the decrease of creatinine clearance. But measurement of creatinine clearance, as derived from 24-hour urine collection, is a cumbersome, impractical, and inaccurate test. The estimated glomerular filtration rate (eGFR) is more accurate and significantly easier to obtain as it is calculated from serum creatinine, age, gender, and ethnicity using the modification of diet in renal disease (MDRD) calculation [30] or the very simple Cockcroft-Gault formula: (140 − number years of age) × Kg body weight/72/mg% of serum creatinine; in females the result × 0.85 [31]. Moderately decreased renal function is defined as eGFR 30–60 mL/min (renal insufficiency).

In some cases, CIN may cause a more severe impairment of renal function with oliguria (<400 mL/24 hrs), requiring dialysis. In these cases the mortality is much higher. Permanent severe renal failure requiring dialysis has been shown to occur in up to 10% of patients with preexisting renal failure who develop further reduction in renal function after coronary angiography [32] or in <1% of all patients undergoing percutaneous coronary intervention using contrast agents [33].

The management of CIN is the same as that for acute renal failure due to other causes [23].

In a prospective study examining the incidence of CIN in a relatively young (54 ± 14) outpatient cohort undergoing contrast-enhanced CT with a low-osmolar, nonionic contrast iopamidol-370, CIN occurred in 11% of patients; it was associated with an increased risk for severe renal failure and death from renal failure [34]. In another prospective, observational study of patients undergoing contrast-enhanced CT, CIN was uncommon among outpatients with mild baseline kidney disease, even without the administration of IV fluids in most patients: <1% of outpatients with GFR >45 mL/min per 1.73 m^2^ manifested an increase in serum creatinine >0.5 mg/dL [35].

In a retrospective study analyzing 11,588 patients who underwent either CT without contrast or CT with a low-osmolar contrast medium (iohexol) or an iso-osmolar contrast medium (iodixanol), no significant difference in the overall incidence of CIN was observed between the iso-osmolar contrast medium and the control groups for all baseline creatinine values. The incidence of acute kidney injury in the low-osmolar contrast medium group paralleled that of the control group up to a creatinine level of 1.8 mg/dL, but increases above this level were associated with a higher incidence of acute kidney injury in the low-osmolar contrast medium group [36].

In a recent retrospective study of CT examinations performed over a 10-year period in 20,242 adult inpatients (10,121 untreated and 10,121 treated with IV contrast media) with sufficient serum creatinine data, in order to determine the effect of IV low-osmolar contrast media (LOCM) on the development of post-CT CIN, stratified by pre-CT eGFR in patients with stable renal function, it has been observed that IV LOCM is a risk factor for nephrotoxicity in patients with a stable eGFR <30 mL/min/1.73 m^2^, with a trend toward significance at 30–44 mL/min/1.73 m^2^, whereas it does not appear to be a nephrotoxic risk factor in patients with a pre-CT eGFR ≥45 mL/min/1.73 m^2^ [37]. Thus, according to these authors, IV LOCM is a nephrotoxic risk factor, but not in patients with a stable serum creatinine level <1.5 mg/dL [38] or eGFR ≥45 mL/min/1.73 m^2^ [37].

In another recent retrospective study involving 53,439 unique patients in whom serum creatinine was regularly checked to determine the causal association and effect of IV iodinated contrast material exposure to the incidence of CIN, it was found that the incidence of CIN was not significantly different between the contrast material group and control group, suggesting that intravenous iodinated contrast media may not be the causative agent in diminished renal function after contrast material administration [39]. In a systematic review and meta-analysis of controlled studies by the same group examining the incidence of CIN in patients exposed to IV contrast medium compared with patients who underwent an imaging examination without contrast medium (control group), a similar incidence of CIN, dialysis, and death was demonstrated between the contrast medium group and control group [40].

The amount of morbidity or mortality observed after CIN that would be avoided if CIN events were prevented remains unknown. A review of observational studies and clinical trials to shed light on the nature of the relationship between CIN and mortality allowed the conclusion that the deaths of some patients with CIN are complicated by factors that cannot be directly related to CIN, such as liver disease, sepsis, respiratory failure, and bleeding. However, it is plausible that CIN contributes to cardiovascular causes of death in patients with CIN [41].

In a 3-year retrospective study in an intensive care unit (ICU), patients undergoing a contrast media-enhanced CT scan in whom changes in serum creatinine between baseline (24 hours before to 12 hours after contrast media injection) and its maximum value over the 96 hours after contrast media injection was recorded (in all 299 patients), the incidence of CIN was 14%. The need for renal replacement therapy and ICU mortality were significantly higher in cases of CIN [42].

Among all procedures utilizing contrast media for diagnostic or therapeutic purposes, coronary angiography and percutaneous coronary interventions (PCI) are associated with the highest rates of CIN [28] mainly related not only to the intra-arterial injection and to the high dosage of the contrast necessary, but also to the type of patients (advanced age, one or more comorbid conditions, and more advanced vascular diseases, such as hypertension and diabetes) [25].

The relationship of CIN to long-term adverse events (e.g., death, stroke, myocardial infarction, end-stage kidney disease, percutaneous coronary revascularization, coronary artery bypass graft surgery, cardiac arrest, development of congestive heart failure or pulmonary edema, and need for permanent pacing) has been studied in 294 patients, with followup of at least 1 year after contrast exposure. The rate of long-term adverse events was higher in individuals with CIN. A reduction in the incidence of CIN and long-term adverse events was observed in regression analyses to adjust for possible known confounders. This supports the view that CIN is causally related to long-term adverse events rates [43].

### 4.1. Pathogenesis

The mechanisms underlying contrast media nephrotoxicity have not been fully elucidated and may be due to several factors. The generally held view is that CIN is caused by a combination of a reduction in medullary blood flow leading to hypoxia and direct tubular damage due to toxicity of contrast media. Hypoxia may lead to the formation of reactive oxygen species (ROS) [44] and it has been argued that these in turn are responsible for contrast media toxicity [45, 46].

The intravenous injection of radiographic contrast medium causes an initial increase in renal blood flow but is then followed by a more prolonged decrease in blood flow and accompanied by a decrease in glomerular filtration rate (GFR), while the extrarenal vessels show transient vasoconstriction followed by decrease in vascular peripheral resistances. The result will be a renal ischaemia, particularly in the medulla (a region already functioning at low oxygen tension under normal physiologic conditions), contributing to the pathogenesis of CIN [47, 48]. Other intrinsic causes of medullary ischemia include increased oxygen consumption, increased intratubular pressure secondary to contrast-induced diuresis, increased urinary viscosity, and tubular obstruction, all frequently associated with dehydration and decrease in the effective intravascular volume [23, 49].

Oxygen delivery to the outer medulla is poor even under normal conditions because of its distance from the descending vasa recta. The majority of* in vitro* experiments carried out to study the effect of contrast media on arteries obtained from different animal species showed differing responses with respect to contraction/dilation depending on the type of vessel and species being studied [8]. Furthermore, the contrast medium was not applied intraluminally, thus precluding any evaluation of the effect of contrast media on the epithelium. However, in one study in which specimens of outer medullary descending vasa recta were isolated from rats and microperfused intraluminally with a buffered solution containing iodixanol, it was demonstrated that contrast media directly constrict descending vasa recta by reducing nitric oxide (NO) and significantly increasing the vasoconstrictor response to angiotensin II, thereby causing local hypoxia [8, 50]. The decrease in NO is believed to be due to its reaction with ROS in particular superoxide [8]. Interestingly, this reaction may lead to the formation of the more powerful oxidant peroxynitrite [51] that may be more detrimental to the physiological milieu.* In vivo* experiments in rats demonstrated that the decrease in cortical and medullary microvascular blood flow induced by contrast medium is partly accounted for by the downregulation of endogenous renal cortical and medullary NO synthesis [52]. To support the role of ROS generated during contrast media administration in vasoconstriction, the use of the superoxide dismutase (SOD) mimetic tempol reduced iodixanol-induced vasoconstriction [50]. More recent work using a recombinant manganese SOD administered* in vivo* to rats undergoing diatrizoate treatment caused an improvement in GFR and a reduction in renal histologic damage [53]. However, the decrease in NO in the vasa recta may not be totally accounted for by increased ROS production as damage to endothelial cells (including apoptosis) may be a factor [8]. Endothelial damage may also release endothelin and hence lead to vasoconstriction [8]. Reduced levels of prostaglandins have also been suggested to predispose to CIN [54].

Direct tubular epithelial cell toxicity by contrast media has been observed in studies of isolated tubule segments and cultured cells substantiated by disruption of cell integrity, the generation of ROS and apoptosis.

As already mentioned, contrast media can cause cellular damage to endothelial cells, being the first to come in contact with intravenously injected contrast agents, but the contrast media are filtered by glomeruli and are concentrated inside the tubules, exposing the tubular cells to even worse direct damage [8].* In vitro* cell culture studies have shown that all types of contrast media cause a decrease in cell viability [55–59]. The biochemical changes underlying these effects have been extended to studying changes in major intracellular signalling pathways involved in cell survival, death, and inflammation [57–60]* in vitro* in cultured human renal tubular cells [61]. Contrast media can cause perturbation of mitochondrial enzyme activity and mitochondrial membrane potential in proximal tubule cell line; in the more distal segments of the kidney, they can cause apoptosis [62]. Studies in animals and* in vitro* studies suggest, in fact, that contrast media can directly induce caspase-mediated apoptosis of renal tubular cells. It seems that contrast-induced apoptosis is due to the activation of shock proteins and the concurrent inhibition of cytoprotective enzymes and prostaglandins [2, 34, 63–67].

Some studies have highlighted the crucial role of mannose-binding lectin (a protein of the complement system) in aggravating the inflammatory response and tissue damage during ischemia/reperfusion injury of several organs, including the kidney. In a clinical trial assessing the importance of serum mannose-binding lectin with respect to the development of CIN, the deficiency of this lectin did not influence the occurrence of CIN as defined by a serum creatinine increment; it was, however, associated with an (limited) increase in cystatin C after the administration of contrast agent [68]. But the increase in serum creatinine after exposure to contrast media is delayed, usually achieving a maximum two to five days after contrast exposure. However, cystatin C, a more sensitive marker, has been shown to rise earlier, to peak as early as 24 hours after contrast administration, thereby detecting even subtle changes in eGFR after acute kidney injury including CIN [69–72]. Thus, in this clinical trial, subjects with mannose-binding lectin deficiency were almost two times less likely to develop an increase of ≥10% in cystatin C after administration of the contrast agent. This suggests that deficiency of mannose-binding lectin might attenuate some of the detrimental effects of contrast media [68].

It has been suggested that an adaptive response to hypoxia in the kidney parenchyma involving the hypoxia-inducible factor (HIF) may play a protective role in the pathogenesis of CIN [54]. Radiocontrast media administered to a rat model of acute renal failure resulted in accumulation of HIF isoforms in the kidney medulla. HIF is a transcription factor that has many targets, one of which is a 32-kilodalton protein, heme oxygenase-1 (HO-1). HO-1 induction results in degradation of prooxidant heme, releasing iron, carbon monoxide (CO), and biliverdin; biliverdin is converted to bilirubin, an antioxidant; iron is sequestered by ferritin; the products of the HO-1 reaction have antioxidant, anti-inflammatory, vasodilatory, and antiapoptotic effects, leading to attenuation of CIN [24].

Whilst the development of iso-osmolar contrast media has been seen as a positive development, the downside is that these tend to be more viscous leading to increased urine viscosity [8]. Increased viscosity may lead not only to greater retention in the kidneys but also to tubular stretching and mechanical stress, causing greater oxidative stress in the thick ascending limbs which may worsen tubular damage. Again, previous pathology in individual patients (e.g., diabetes mellitus and chronic nephropathy) increases the severity of damage, thus enhancing the final risk of CIN [8].

### 4.2. Risk Factors

Identification of patients at high risk for the development of CIN is of major importance.

The European Society of Urogenital Radiology has suggested that the real risks for CIN are represented by preexisting renal impairment particularly secondary to diabetic nephropathy, salt depletion and dehydration, congestive heart failure, age greater than 70 years, and concurrent use of nephrotoxic drugs [27, 73].

But the risk factors for CIN include important conditions that are both modifiable and nonmodifiable (Table 1).

(A) Preexisting impairment of renal function, irrespective of cause: the higher the baseline creatinine value or, better, the lower the eGFR, the greater the risk of CIN. An eGFR of 60 mL/min/1.73 m^2^ is a reliable cut-off point for identifying patients at high risk for the development of CIN. The incidence of CIN in patients with underlying chronic renal failure is extremely high, ranging from 14.8 to 55% [28]. In a retrospective observational in-hospital study, however, it has been recently demonstrated that CIN occurred with similar frequency, following coronary angiography, in both patients with and without chronic kidney disease [74].

(B) Diabetes mellitus particularly when associated with renal insufficiency [75]: diabetes predisposes to CIN. At any given degree of baseline GFR, diabetes doubles the risk of developing CIN compared with nondiabetic patients. The incidence of CIN in diabetic patients varies from 5.7 to 29.4% [28]. The administration of iodinated radiocontrast media to diabetics acutely reduces renal parenchymal oxygenation, a reduction that is most prominent in the renal medulla, already functioning at low oxygen tension [76]. The biologically active endothelins are produced by proteolysis of the precursor preproendothelins under the action of endothelin-converting enzyme that plays a key role in the rising circulating and renal endothelin levels found in diabetes and after exposure to contrast agents. This may explain the particular susceptibility of diabetic patients to contrast media [76]. The high incidence of CIN in diabetic patients has also been attributed to hypersensitivity of renal vessels of diabetics to adenosine, a vasoconstrictive agent [77]. It has been demonstrated that, in patients with diabetes, hypercholesterolemia is the strongest predictor of CIN [78]. Despite the mentioned evidence, most authors do not regard the presence of diabetes mellitus in the absence of renal failure as a risk factor for CIN. In diabetic patients with preserved renal function and without other risk factors, in fact, the incidence of CIN is comparable to that of a nondiabetic population [79]. But an established observation is that the coupling of chronic kidney disease and diabetes dramatically increases the risk for CIN compared with that observed for chronic kidney disease alone [80]. It has been observed, in fact, that diabetic patients with advanced chronic renal failure (serum creatinine >3.5 mg/dL) due to causes other than diabetic nephropathy are at significantly higher risk for CIN [81].

(C) Concomitant use of nephrotoxic drugs such as aminoglycosides, cyclosporin A, amphotericin, and cisplatin: also the concomitant use of nonsteroidal anti-inflammatory drugs represents an important risk factor because of their inhibition of the vasodilatory prostaglandins [82, 83].

(D) Concomitant use of angiotensin-converting enzyme inhibitors or angiotensin receptor blockers: the role of renin-angiotensin-aldosterone system blocking agents such as angiotensin-converting enzyme inhibitors (ACEIs) and angiotensin II receptor blockers (ARBs) in the pathophysiology of CIN remains controversial. There are conflicting opinions on this point [84]. Angiotensin II is a main effector peptide in the renin-angiotensin system and plays a very important role in controlling renal homeostasis as a vasoconstrictor. According to most authors, patients with chronic renal disease under treatment with ACEIs or ARBs are at higher risk for developing CIN [74, 85–89] particularly in the elderly [90]. According to other investigators, captopril prevents CIN in diabetic patients by reducing the increase of angiotensin II [91]. In animal models, in fact, it has been demonstrated that telmisartan protects the renal tissue from nephrotoxicity of contrast media [92]. Some authors suggest discontinuing the use of ACEIs and ARBs 48 hours prior to exposure to radiocontrast agents, especially in patients with multiple risk factors [89]. According to others, withholding ACEIs and ARBs 24 h before coronary angiography does not appear to influence the incidence of CIN in stable patients with chronic renal failure [93]. According to KDIGO guidelines for Acute Kidney Injury Work Group, there is insufficient evidence to recommend discontinuation of these medications prior to contrast administration [94].

(E) Reduction of effective intravascular volume: this may be due to congestive heart failure, liver cirrhosis, or salt depletion secondary to abnormal fluid losses associated with insufficient salt intake.

(F) Severe dehydration causes reduction of effective intravascular volume. Furthermore, renal vasoconstriction induced by adenosine is accentuated during sodium depletion and is reduced during volume expansion [48].

(G) Prolonged hypotension [23, 95].

(H) Multiple myeloma: CIN was described for the first time in a patient with multiple myeloma receiving intravenous pyelography [96]. Early articles linked IV administration of contrast agents with the development of renal failure in patients with multiple myeloma, leading to the conclusion that IV contrast media are contraindicated in patients with myeloma because they are at a high risk for developing CIN [97–101]. A recent retrospective clinical study examined the risk of CIN in patients with multiple myeloma following nonionic iodinated contrast media injection during CT. On the basis of their results the authors concluded that the incidence of CIN in patients with multiple myeloma with a normal serum creatinine is low and correlates with *β*_2_-microglobulin levels; thus, the administration of contrast agents in these patients is relatively safe [102]. The mean *β*_2_-microglobulin serum level (which increases with both higher tumor burden and decreased renal function) did display a statistically significant association with CIN development. According to the authors, a review of *β*_2_-microglobulin serum levels may be beneficial before administering IV contrast agent to patients with myeloma, because it likely serves as a marker of patients who are at a higher risk of developing renal insufficiency and CIN. They suggested a threshold value of less than 2.8 mg/L of *β*_2_-microglobulin serum level for essentially eliminating the risk of CIN [102]. In multiple myeloma CIN has been attributed to precipitation of radiographic contrast media molecules with Tamm-Horsfall proteins and other abnormal proteins, and tubular epithelial cells damaged and desquamated due to ischemia [23, 103].

(I) Use of large doses of contrast media and their multiple injections within 72 hrs [23, 104, 105]: the risk of CIN is dose-dependent; it increases with the volume of contrast medium administered during the procedure [33, 106, 107]. Larger volumes of contrast agents are used in coronary angiography than in other imaging studies. Therefore, patients who have coronary angiography (who usually have one or more comorbid conditions) have CIN more frequently than other patients [108, 109].

(J) Route of administration: many studies have demonstrated that intravenous contrast media are less risky than intra-arterial contrast media [26, 110–114]. Radiographic contrast media are more nephrotoxic when given intra-arterially because of the higher acute intrarenal concentration [23, 115], particularly if the arterial injection is suprarenal [116]. It has been demonstrated that, while performing aortography, the closer to the renal arteries the injection of contrast medium occurs, the higher the risk of CIN is [117].

(K) Osmolality of contrast media compared with the osmolality of plasma seems to play an important role in nephrotoxicity. Contrast media usually have high viscosity and greater osmolality (more molecules per kilogram of water) than plasma. Ionicity is the characteristic of a molecule to break up into a cation and an anion, resulting in more molecules per kilogram of water and thus increasing osmolality. Nonionic agents not having this property are less osmolar. The osmotoxic effect of contrast agents is described in terms of the ratio of iodine atoms to dissolved particles: the higher the ratio, the better the attenuation of X-rays [23, 118]. Ionic high-osmolar contrast media (HOCM, e.g., diatrizoate, 1500 to 1800 mOsm/kg, i.e., 5–8 times the osmolality of plasma) have a ratio of 1.5 : 1, nonionic low-osmolar contrast media (LOCM, e.g., iohexol, 600 to 850 mOsm/kg, i.e., 2-3 times the osmolality of plasma) have a ratio of 3 : 1, and nonionic iso-osmolar contrast media (IOCM, e.g., iodixanol, approximately 290 mOsm/kg, i.e., the same osmolality as plasma) have a ratio of 6 : 1 [119]. Adverse reactions to contrast media range from 5% to 12% for HOCM and from 1% to 3% for LOCM. It has been shown that LOCM rather than HOCM are beneficial in the prevention of CIN to patients with preexisting renal failure [118, 120–122]. Iodixanol (IOCM) seems less nephrotoxic than iohexol (LOCM) [118, 123] at least in patients with intra-arterial administration of the drug and renal insufficiency [115, 124]. But recent studies and meta-analyses have found no significant difference in the rates of CIN between IOCM and LOCM [124–127].

(L) Advanced age (>65 years): the reasons for higher CIN risk in the elderly are multifactorial, including age-related changes in renal function and the presence of old vessels, of coronary artery disease [28, 104].

(M) Anemia: anemia is a risk factor for CIN by contributing to renal ischemia. It has been demonstrated that a low hematocrit is an important risk factor for CIN. The rates of CIN were the highest (28.8%) in patients who had the lowest level for both baseline eGFR and hematocrit. Patients with the lowest eGFR but relatively high baseline hematocrit values had remarkably lower rates of CIN [128].

(N) Advanced congestive heart failure or compromised left ventricle systolic performance has been stated to be important risk factors for CIN [28].

(O) Sepsis is a risk factor, probably because of direct tubular damage by bacterial toxins and impairment of circulation [23, 83, 104].

(P) Renal transplant: patients with renal transplantation are at a higher risk of CIN due to concomitant use of nephrotoxic drugs, such as cyclosporine, and higher prevalence of diabetes and renal insufficiency. In a retrospective study, the incidence of CIN in renal allograft recipients was lower, 15.3%, in patients who received intravenous hydration compared to 42.8% in patients who received no prophylaxis prior to intravenous or intra-arterial contrast studies (coronary angiogram, CT scan with intravenous contrast, angiogram for evaluation of peripheral vascular disease, allograft angiogram with angioplasty, pulmonary angiogram, and intravenous pyelogram) [129].

### 4.3. Prevention

In patients at high risk of CIN, renal function should be carefully monitored by measuring serum creatinine and calculating the eGFR before and once daily for 5 days after the radiographic procedure [23].

Appropriate procedures to prevent CIN include the following.

(1) Adequate hydration of the patient: it has been stated that volume supplementation is the cornerstone for the prevention of CIN, being safe, effective, and inexpensive [130]. On a theoretical basis, hydration causes expansion of intravascular volume, thereby suppressing the renin-angiotensin cascade and consequently reducing renal vasoconstriction and hypoperfusion; the result is an increase of diuresis, thereby limiting the duration of contrast material contact with renal tubules and therefore its toxicity on tubular epithelium [131, 132]. There is evidence to support hydration as a preventative measure in patients at high risk for CIN [133]. The suggestion to limit fluid intake starting the day before contrast administration should be abolished. On the contrary, if there is no contraindication to oral ingestion, fluid intake should be encouraged, for example, 500 mL of water or soft drinks (tea, mineral water) orally before and 2,500 mL for 24 hours after contrast administration; this fluid intake should secure diuresis of at least 1 mL/min in a nondehydrated patient [134]. High-risk patients should be administered 0.9% saline by IV infusion at a rate of approximately 1 mL/kg per hour, beginning 6–12 hours before the procedure and continuing for up to 12–24 hours after the radiographic examination, if diuresis is appropriate and cardiovascular condition allows it [23, 130]. Several studies have confirmed the efficacy of adequate hydration with IV infusion of normal saline solution (NaCl, 0.9%) or a half-strength saline solution (NaCl, 0.45%) [134, 135]. However, the occurrence of CIN was significantly reduced with isotonic (0.7%) versus half-isotonic volume supplementation (2.0%) [130]. It has also been suggested and/or demonstrated by clinical studies or meta-analysis that sodium bicarbonate hydration is superior to sodium chloride hydration [136–142]. Most recent meta-analysis found bicarbonate effective only with LOCM and not IOCM and with the greatest effect for urgent coronary procedures [143]. For patients undergoing an emergency coronary angiography or intervention, 154 mEq/L infusion of sodium bicarbonate has been used, as a bolus of 3 mL/kg/hour for 1 hour before the administration of contrast, followed by 1 mL/kg/hour for 6 hours during and after the procedure [137]. In a double-blind and randomized clinical trial, the risk of CIN was significantly lower in patients undergoing coronary angiography or percutaneous coronary intervention pretreated with bicarbonate infusion in combination with oral acetazolamide, a carbonic anhydrase inhibitor [144, 145]. In one paper sodium citrate was used to alkalinize the urine [146]. It seems, in fact, that a direct causal relationship exists between low pH of tubular fluid and enhanced activity of generated ROS to damage renal tubular cells. Sodium bicarbonate (that is excreted with urine following either bicarbonate infusion or acetazolamide administration) decreases the acidification of urine and medulla, thereby reducing the production and increasing the neutralization of oxygen free radicals; this protects the kidney from injury by contrast agents [139, 140, 147, 148]. Other investigators did not find a benefit when hydration with sodium bicarbonate was compared to hydration with sodium chloride for the prevention of CIN in patients with moderate to severe chronic kidney disease who were undergoing coronary angiography [149–152]. Some authors have also found that the use of intravenous sodium bicarbonate was associated with even an increased incidence of CIN that might be due to its prooxidant properties (bicarbonate in the presence of ROS would enhance the generation of ROS) [153]. This is the European Renal Best Practice position:* “We recommend volume expansion with either isotonic sodium chloride or sodium bicarbonate solutions, rather than no volume expansion, in patients at increased risk for CIN”* [154]. A randomized, double-blind, multicenter trial, prevention of serious adverse events following angiography (PRESERVE), is currently underway enrolling 8,680 high-risk patients undergoing coronary or noncoronary angiography, to compare the effectiveness of IV isotonic sodium bicarbonate versus IV isotonic sodium chloride and oral N-acetylcysteine versus oral placebo for the prevention of serious, adverse outcomes associated with CIN [155].

(2) Discontinuation of nephrotoxic drugs: potentially nephrotoxic medications, such as aminoglycosides, vancomycin, amphotericin B, and nonsteroidal anti-inflammatory drugs, should be discontinued before contrast media administration [48]. Sometimes the use of aminoglycosides is absolutely necessary; in these cases it is recommended that they are used for as short a period of time as possible. The European Renal Best Practice [154] position is:* “We suggest not using more than one shot of aminoglycosides for the treatment of infections… We recommend that, in patients with normal kidney function in steady state, aminoglycosides are administered as a single-dose daily rather than multiple-dose daily treatment regimens…* (meanwhile)* monitoring aminoglycoside drug levels”*. Concerning the use of amphotericin B, ERBP recommends that saline loading should be implemented in all patients receiving any formulation of amphotericin B [154]. Metformin is an oral antihyperglycemic medication widely used to treat type 2 diabetes, is eliminated unchanged primarily via the kidneys (>90% within 24 hours), and, as a biguanide, stimulates intestinal production of lactic acid. If contrast media administration causes renal failure, metformin is not excreted and can reach toxic levels resulting in lactic acidosis with a significant associated mortality (~50%). Thus, it is recommended that metformin be discontinued at least 12 hours before the contrast administration and not be resumed for a minimum of 36 hours after the procedure, or longer if the serum creatinine has not returned to baseline [3].

(3) Use of N-acetylcysteine: as already mentioned, experimental findings* in vitro* and* in vivo* have demonstrated enhanced hypoxia and the formation of ROS within the kidney following the administration of iodinated contrast media; this may play a role in the development of CIN. Since ROS have been suggested to play a crucial role in renal damage by contrast agents, the antioxidant N-acetylcysteine has been thought to act either as a free-radical scavenger or as a reactive sulfhydryl compound as well as a factor able to increase the vasodilating effect of NO [23, 45, 156]. Short-duration pretreatment with N-acetylcysteine significantly reduced contrast-medium-induced cytotoxicity in human embryonic kidney cells treated with three different contrast media: ionic ioxitalamate, nonionic low-osmolar iopromide, and iso-osmolar iodixanol [157]. N-acetylcysteine has been shown to ameliorate ischemic renal failure in animal models [158]. But the protective effect of N-acetylcysteine against CIN in high-risk patients is still controversial. Some authors have reported protective effect [159–161]; others have denied it [153, 162–168]. It has also been suggested that N-acetylcysteine would have additional benefits, other than its reported renoprotective effects, on heart and vessels [169]. Despite these controversial results, the use of N-acetylcysteine in high-risk patients, with an oral dose of 600 mg twice daily on the day before and the day of procedure, has been suggested [23]. The use of IV doses of 150 mg/kg over half an hour before the procedure or 50 mg/kg administered over 4 hours has been suggested for patients unable to take it orally [160].

(4) Use of antioxidant vitamin C (ascorbic acid): in an* in vitro* study ascorbic acid did not reduce the contrast-medium-induced cytotoxicity in human embryonic kidney cells treated with three different contrast media: ionic ioxitalamate, nonionic low-osmolar iopromide, and iso-osmolar iodixanol [157]. Conflicting results have been obtained with the clinical use of ascorbic acid in the prevention of CIN. Ascorbic acid (3 g given orally 2 hours before the procedure and 2 g in the night and the morning after the procedure) had a protective effect on the kidney in patients undergoing coronary angiography and/or intervention [170, 171]. Other investigators have denied the prophylactic use of ascorbic acid in patients with renal dysfunction exposed to contrast dye [172]. High-dose N-acetylcysteine (1,200 mg orally twice on the day before and the day of coronary catheterization) seems more beneficial than ascorbic acid in preventing CIN, especially in diabetic patients with renal insufficiency undergoing coronary angiography [173].

(5) Use of the antioxidant vitamin E (*α*- or *γ*-tocopherol):* in vivo* and* in vitro* studies have already demonstrated the antioxidative and anti-inflammatory properties of vitamin E. Prophylaxis oral administration with either 350 mg/day of *α*-tocopherol or 300 mg/day of *γ*-tocopherol (5 days prior to the coronary procedure, continuing for a further 2 days after procedure) in combination with 0.9% saline (at a rate of 1 mL/kg/h for 12 hours before and 12 hours after elective coronary procedures) has been shown to be effective in protecting against CIN in chronic kidney disease patients undergoing elective coronary procedures with the low-osmolar, nonionic contrast medium iopromide: CIN developed in 14.9% of patients in the placebo group, but only in 4.9% and 5.9% in the *α*- and *γ*-tocopherol groups, respectively [174].

(6) Use of statins: hypercholesterolemia has been suggested as a predisposing factor to CIN on the basis of a study in experimental AKI, characterized by compromised NO synthesis and enhanced ROS generation [175]. Recent studies have shown a beneficial effect of the use of statins to prevent CIN in patients undergoing percutaneous coronary intervention [176–178]. In a recent study on 2998 patients with diabetes mellitus and chronic kidney disease undergoing arterial contrast media injection for coronary/peripheral arterial angiography, rosuvastatin, given at a dosage of 10 mg/day (*n* = 1,498) for five days (two days before and three days after procedure), significantly reduced the risk of CIAKI [179]. The results of experimental studies in rats have shown that simvastatin dose-dependently attenuated contrast-induced rise of creatinine, urea, and structural abnormalities suggesting its nephroprotective effect [180]. Chronic pravastatin treatment before contrast media exposure was important for preventing CIN in patients with renal insufficiency undergoing coronary angiography or percutaneous coronary intervention [181]. Patients on pravastatin had a significantly lower incidence of CIN compared to patients on simvastatin [182]. The use of short-term atorvastatin (40 mg/day started 3 days before coronary angiography) and chronic statin therapy may have a role in protecting renal function after elective coronary angiography: after coronary angiography, serum creatinine and eGFR were significantly better in patients using atorvastatin than in controls [183]. Along with lowering serum cholesterol, statins have pleiotropic effects in the vasculature, by decreasing endothelin synthesis, decreasing inflammation and improving endothelial function, and reversing contrast-induced oxidative stress. The nephroprotective effect of statins against contrast media might not involve lipid metabolism [184]; it can be attributed mainly to its antioxidant, anti-inflammatory, and antithrombotic properties and to its vasodilator properties, mediated by NO, that improve renal microcirculation [180]. On the basis of recent literature, many authors support the utilization of statins as adjuvant pharmacologic therapy before percutaneous coronary intervention [176, 177, 185]. A meta-analysis supports the effectiveness of short-term high-dose statin pretreatment for both decreasing the level of serum creatinine and reducing the rate of CIN in patients undergoing diagnostic and interventional procedures requiring contrast media [186]. Patients with acute coronary syndrome undergoing percutaneous coronary intervention were given short-term pretreatment with high-dose atorvastatin load (atorvastatin 80 mg 12 hours before intervention with another 40 mg before procedure, followed by long-term atorvastatin treatment 40 mg/day); this treatment prevented CIN and shortened hospital stay [187].

(7) Use of nebivolol: nebivolol is a *β*1-adrenergic receptor antagonist, a third-generation beta-adrenergic blocker, with vasodilator and antioxidant properties [188, 189]. It has been hypothesized that nebivolol may protect the kidney against CIN through its antioxidant and NO-mediated vasodilator action. An experimental study in rats has demonstrated the protective role of nebivolol against CIN [190]. In fact it decreased the medullar congestion, protein casts, and tubular necrosis which occurred secondary to contrast media, decreased the systemic and renal oxidative stress which occurred after contrast media administration, decreased the microproteinuria which occurred secondary to contrast media, and increased the kidney nitrite level which was decreased by contrast media [190]. Based on this study on nebivolol and CIN, it was hypothesized that nebivolol may also protect the kidney in humans against CIN through its antioxidant and NO-mediated vasodilator actions. The use of oral nebivolol for one week at a dose of 5 mg per day decreased the incidence of contrast-induced nephropathy in patients with renal dysfunction who underwent coronary angiography [191]. Pretreatment with nebivolol (5 mg nebivolol every 24 hours for 4 days) appeared to be protective against nephrotoxic effects of contrast media in human beings subjected to iodinated contrast agent for coronary angiography and ventriculography [192].

(8) Use of human serum albumin-thioredoxin (HSA-Trx) (still experimental): thioredoxin-1 (Trx) is a ubiquitous low-molecular-weight protein, produced in the human body in response to oxidative stress conditions. It has a protective effect against oxidative stress being a ROS scavenger, with a very short half-life because of its fast elimination by the kidney [193]. An HSA-Trx fusion protein has recently been obtained with a half-time 10 times longer than that of thioredoxin in normal mice.* In vivo* (in rats) and* in vitro* (on human proximal tubular cells) studies have demonstrated its ROS scavenging activity. Thus HSA-Trx prevents CIN and renal tubular apoptosis, via its extended antioxidative action, in a rat model of ioversol-induced CIN [193].

(9) Use of sodium butyrate (still experimental): it has been recently demonstrated in rats that sodium butyrate possesses anti-inflammatory activities; it decreases the activation of nuclear factor kappa B (NF-*κ*B), thereby reducing inflammation and oxidative damage in the kidney of rats subjected to CIN [194].

(10) Use of high doses of steroids: it has been recently suggested that high-dose steroids (oral prednisone, 1 mg/kg, 12–24 hours before the angiographic procedure, at 6 am on the day of the procedure, and 24 hours after the procedure) given concurrently with IV saline (12 hours before the procedure, at an infusion rate of 1 mL/kg per hour of 0.9% saline) may be useful for protecting the renal tubule during diagnostic/interventional endovascular procedures involving the arterial administration of iodinated contrast media (either iso-osmolar iodixanol or low-osmolar iohexol) [195]. The rationale is that steroids may have a favorable impact on inflammation and renal tubular cell apoptosis and necrosis, as observed in models of renal ischemia-reperfusion in which dexamethasone had a protective effect against injury [196].

(11) Choice of contrast medium: (Table 2) low-osmolar contrast media (LOCM, e.g., iohexol) are less nephrotoxic than high-osmolar contrast media (HOCM, e.g., diatrizoate), but iso-osmolar contrast media (IOCM, e.g., iodixanol) seem to be even less so [23]. In human prospective trials and meta-analysis, no statistical significant difference in nephrotoxicity between high-osmolar (HOCM) and nonionic low-osmolar (LOCM) contrast media has been found in patients with normal renal function. However, in patients with chronic kidney disease, there is a higher incidence of CIN with high-osmolar contrast media (HOCM) than with the nonionic, low-osmolar contrast media (LOCM) [113, 197].

When the cytotoxicity of LOCM and IOCM was evaluated on human embryonic kidney cells, porcine proximal renal tubular cells, and canine Madin-Darby distal tubular renal cells, both LOCM and IOCM induced a dose-dependent renal cell apoptosis that was prevented by N-acetylcysteine and ascorbic acid [198].

A recent analysis including 36 randomized controlled trials for a total of 7166 patients (3672 patients receiving the IOCM iodixanol and 3494 patients receiving other LOCMs) showed CIN incidence for iodixanol not statistically different from the pooled LOCM; a significant reduction of CIN with iodixanol was only observed in direct comparison of iodixanol with the LOCM iohexol [199]. This suggests that there are differences among low-osmolar contrast media (LOCM) such that each molecule should be considered individually.

A multicenter, randomized, double-blind comparison of iopamidol (LOCM) and iodixanol (IOCM) has been performed in patients with chronic kidney disease (eGFR between 20 and 59 mL/min) who underwent cardiac angiography or percutaneous coronary interventions. Serum creatinine (SCr) levels and eGFR were assessed at baseline and 2 to 5 days after receiving the contrast agents. The primary outcome was a postdose SCr increase ≥0.5 mg/dL over baseline. Secondary outcomes were a postdose SCr increase ≥25%, a postdose eGFR decrease of ≥25%, and the mean peak change in SCr. The rate of CIN was not statistically different after the intra-arterial administration of iopamidol or iodixanol to high-risk patients, with or without diabetes mellitus. Any true difference between the agents was small and not likely to be clinically significant [125].

Thus, a number of prospective clinical trials and meta-analyses have confirmed that isosmolar (IOCM) and low-osmolar contrast media (LOCM) have similar safety profiles, with rare exceptions (see most recent meta-analysis by Biondi-Zoccai) [200].

(12) Use of lowest dosage of contrast media: the development of newer imaging technologies has facilitated faster image acquisition; this has enabled radiologists to perform studies with less intravascular contrast, because the duration of time over which contrast needs to be administered has shortened [201]. Considering that high doses of contrast media are required for percutaneous coronary intervention, several formulas have been suggested to calculate the dosage that is least dangerous for renal function. Cigarroa's formula suggests the following contrast material limit: 5 mL of contrast per kilogram body weight/serum creatinine (mg/dL) with maximum dose acceptable of 300 mL for diagnostic coronary arteriography [202]. Laskey's formula suggests the volume of contrast to calculated creatinine clearance ratio with a cut-off point for the ratio at 3.7 for percutaneous coronary intervention: a ratio >3.7 would be associated, following contrast use, with a decrease in creatinine clearance [203] and a significant increase in mortality of patients with ST elevation myocardial infarction [204]. More recently the cut-off point for Laskey's formula has been placed at 2.0: below a ratio of 2.0 CIN would be a rare complication of percutaneous coronary intervention, but it would increase dramatically at a ratio of 3.0 [201, 205]. Other authors have suggested using the ratio of grams of iodine to the calculated creatinine clearance; a ratio 1.42, or better 1.0, would prevent CIN. But the different results obtained by different authors suggest that this needs to be validated further before being accepted in clinical practice, considering also that patients are not a homogeneous group, since some of them may have complications such as hypotension, shock, and reduced left ventricular systolic function that are themselves risks for CIN [201].

(13) Use of furosemide or mannitol associated with saline infusion (to prevent salt depletion) has been thought to be beneficial in protecting against CIN since enhanced transport activity with oxygen consumption is a principal cause of renal hypoxia and both furosemide and mannitol reduce transport activity; but several studies have demonstrated either no effect or even deleterious effect on renal function [135, 206, 207]. The administration of furosemide and mannitol should be avoided [134, 208]. Diuretics should be avoided in high-risk patients who are susceptible to volume depletion before contrast exposure [74].

(14) Use of atrial natriuretic peptide (ANP) has also failed to protect against CIN [207, 209].

(15) Use of calcium channel blockers: calcium ions seem to play a role in the pathogenesis of CIN. Ca^2+^ overload is considered to be a key factor in CIN. The Na^+^/Ca^2+^ exchanger system is one of the main pathways of intracellular Ca^2+^ overload. It has been demonstrated that in rats the pretreatment with KB-R7943, an inhibitor of the Na^+^/Ca^2+^ exchanger system, significantly and dose-dependently suppressed the increase of serum creatinine following diatrizoate administration [210]. The increase in intracellular calcium provokes a vasoconstrictive response in intrarenal circulation and would be an important mediator of epithelial cell apoptosis and necrosis. Measures have been used to reduce the entry of calcium ions into the animal's cells to prevent the reduction in renal blood flow and GFR secondary to vasoconstrictor stimuli [48]. Since hypoperfusion and hypoxia aggravate cytotoxic cell damage and oxidative stress, means of improving renal perfusion are likely to diminish tissue damage. Thus, theoretically, calcium channel blockers would have protective effects, but their use has given controversial results. Some authors have observed protective effects against CIN [211, 212]; others have denied any beneficial results [135, 213, 214].

(16) Use of adenosine antagonists (theophylline and aminophylline) (based on the observation that urinary adenosine is increased after contrast medium administration) has also given controversial results. Some authors have observed protective effect against CIN [215–219]; others have denied any beneficial results [220, 221]. Considering their side effects, it has been stated that adenosine antagonists cannot yet be recommended for routine prophylactic use of CIN [23].

(17) Use of dopamine agonists (fenoldopam and fenoldopam mesylate) to prevent CIN has been disappointing. Despite benefits demonstrated in some studies [222–224], many studies have shown no protective effects against CIN [163, 207, 221, 225].

(18) Use of endothelin receptor blockers: plasma and urine endothelin-1 levels rise in diabetes and after exposure to high doses of contrast media suggesting a role of endothelin-1 in diabetic nephropathy and in CIN [48, 76]. Endothelin receptor blockers, however, have been proven deleterious as a prophylactic tool against CIN [226].

(19) Use of prostaglandin E1 has given some positive protective results on renal function following contrast medium injection in patients with preexisting renal impairment [227].

(20) Haemodialysis or haemofiltration has been used to remove radiocontrast media immediately after the radiographic procedure. However, extracorporeal removal of contrast media does not decrease the incidence of acute renal failure in high-risk patients [228–231]. European Renal Best Practice [154] position (2012):* “We do not recommend using prophylactic intermittent haemodialysis (IHD) or haemofiltration* [217]* for the purpose of prevention of CIN”* [154].

Use of magnetic resonance imaging (MRI) with gadolinium-based contrast agent. According to KDIGO guidelines for Acute Kidney Injury Work Group,* “Consider alternative imaging methods in patients at increased risk for CI-AKI”* [94]. MRI may be an alternative imaging method. Gadolinium is a paramagnetic metal ion. Gadolinium-based contrast agents are used in imaging as a contrast agent in MRI and in magnetic resonance angiography [224]. They are not iodinated compounds; hence no risk for nephropathy is expected [232]. It has been reported, however, that the use of gadolinium-based contrast agents may be associated with acute renal failure or nephrogenic systemic fibrosis (rare pathology that causes fibrosis of the skin and connective tissues throughout the body involving several organs, with kidney included) particularly in patients with preexisting renal failure [233, 234].

## 5. Conclusions

- The first rule to follow in patients at risk of CIN is monitoring renal function by measuring serum creatinine and calculating the eGFR before and once daily for 5 days after the radiographic procedure.
- Potentially nephrotoxic medications, such as aminoglycosides, vancomycin, amphotericin B, metformin, and nonsteroidal anti-inflammatory drugs, should be discontinued before contrast media administration. If the use of aminoglycosides is absolutely necessary, avoid using more than one shot of aminoglycosides for the treatment of infections.
- In the choice of the contrast agent, either IOCM or LOCM should be preferred.
- Use the lowest dosage of contrast media.
- Fluid intake should be encouraged, for example, 500 mL of water or soft drinks (tea, mineral water) orally before and 2,500 mL for 24 hours after contrast administration. High-risk patients should be administered 0.9% saline by IV infusion at a rate of approximately 1 mL/kg per hour, beginning 6–12 hours before the procedure and continuing for up to 12–24 hours after the radiographic examination, if diuresis is appropriate and cardiovascular condition allows it.
- In high-risk patients N-acetylcysteine may also be given with an oral dose of 600 mg twice daily on the day before and the day of procedure. For patients unable to take it orally, IV doses of 150 mg/kg over half an hour before the procedure or 50 mg/kg administered over 4 hours may be used.

Research is still in progress on the protective effects of some drugs on radiocontrast media, as mentioned above, whose toxicity's mechanisms are very complex and not well known yet [235].

## Figures and Tables

**Table 1:** Risk factors for the development of contrast-induced nephropathy (CIN).

Nonmodifiable risk factors	Modifiable risk factors
Advanced age (>65 years)	Large doses and multiple injections of contrast media
Preexisting impairment of renal function	Route of administration
Advanced congestive heart failure	Osmolality of contrast media
Diabetes mellitus	Severe dehydration
Multiple myeloma	Prolonged hypotension
Sepsis	Anemia
Compromised left ventricle systolic performance	Reduction of effective intravascular volume
Renal transplant	Concomitant use of nephrotoxic drugs
	Concomitant use of ACEi and/or ARBs

Abbreviations: ACEi: angiotensin-converting enzyme inhibitors; ARBs: angiotensin II receptor blockers.

**Table 2:** Iodinated contrast media commonly used in clinical practice.

Name	Type	Iodine content (mg/mL)	mOsm/kg	Osmolality type
Ionic				
Diatrizoate (Hypaque 50)	Monomer	300	1,550	HOCM
Metrizoate Isopaque (Conray 370)	Monomer	370	2,100	HOCM
Ioxaglate (Hexabrix)	Dimer	320	580	LOCM
Nonionic				
Iopamidol (Isovue-370)	Monomer	370	796	LOCM
Iohexol (Omnipaque 350)	Monomer	350	884	LOCM
Iodixanol (Visipaque 320)	Dimer	320	290	IOCM

Ionic and nonionic contrast media may be monomeric or dimeric; 3 iodine atoms are present on each benzene ring of the contrast medium: if a contrast molecule contains only 1 benzene ring, it is called a monomer; if it contains 2 benzene rings, it is called a dimer. In solution, ionic contrast media break up into their anion and cation components, thereby increasing osmolality, while nonionic contrast media do not break up in solution. Nonionic dimers are the ideal contrast media as they deliver the most iodine with the least effect on osmolality.

The osmolality of contrast media is compared with the osmolality of plasma. HOCM: high-osmolar contrast media have the highest osmolality, that is, 5–8 times the osmolality of plasma. LOCM: low-osmolar contrast media have an osmolality still higher than plasma, that is, 2-3 times the osmolality of plasma. IOCM: iso-osmolar contrast media have the same osmolality as plasma.
